# Hcfc1a regulates neural precursor proliferation and *asxl1* expression in the developing brain

**DOI:** 10.1186/s12868-020-00577-1

**Published:** 2020-06-10

**Authors:** Victoria L. Castro, Joel F. Reyes, Nayeli G. Reyes-Nava, David Paz, Anita M. Quintana

**Affiliations:** Department of Biological Sciences and Border Biomedical Research Center, The University of Texas at El Paso, El Paso, TX 79968 USA

**Keywords:** *HCFC1*, Neural precursor cells (NPCs), Brain development, *asxl1*

## Abstract

**Background:**

Precise regulation of neural precursor cell (NPC) proliferation and differentiation is essential to ensure proper brain development and function. The *HCFC1* gene encodes a transcriptional co-factor that regulates cell proliferation, and previous studies suggest that HCFC1 regulates NPC number and differentiation. However, the molecular mechanism underlying these cellular deficits has not been completely characterized.

**Methods:**

Here we created a zebrafish harboring mutations in the *hcfc1a* gene (the *hcfc1a*^co60/+^ allele), one ortholog of *HCFC1*, and utilized immunohistochemistry and RNA-sequencing technology to understand the function of *hcfc1a* during neural development.

**Results:**

The *hcfc1a*^co60/+^ allele results in an increased number of NPCs and increased expression of neuronal and glial markers. These neural developmental deficits are associated with larval hypomotility and the abnormal expression of *asxl1*, a polycomb transcription factor, which we identified as a downstream effector of *hcfc1a*. Inhibition of *asxl1* activity and/or expression in larvae harboring the *hcfc1a*^co60/+^ allele completely restored the number of NPCs to normal levels.

**Conclusion:**

Collectively, our data demonstrate that *hcfc1a* regulates NPC number, NPC proliferation, motor behavior, and brain development.

## Background

Neural precursor cells (NPCs) give rise to the differentiated cells of the central nervous system and defects in the number produced, their proliferation, and/or survival can result in a variety of neural developmental disorders. These disorders include intellectual disability [1], cognitive dysfunction [2], behavioral impairment [3], microcephaly [4], epilepsy [5], autism spectrum disorders [6], and cortical malformations [7]. Previous studies have demonstrated a complex network of transcription factors that are responsible for modulating NPC function including SOX2 [8], SOX1 [9], NESTIN [10], and PAX transcription factors [11]. Recent evidence suggests that the *HCFC1* gene, which encodes a transcriptional cofactor, is essential for stem cell proliferation and metabolism [12, 13] in a variety of different tissue types, including NPCs [14–17]. These data strongly suggest that HCFC1 is part of a more global transcriptional program modulating NPC proliferation and differentiation.

HCFC1 regulates a diverse array of target genes and has been shown to bind to the promoters of more than 5000 unique downstream target genes [18]. Consequently, the molecular mechanisms by which HCFC1 regulates NPC proliferation and differentiation are complex. Mutations in *HCFC1* cause methylmalonic acidemia and homocysteinemia, cblX type (*cblX*) (309541). *cblX* is an X-linked recessive disorder characterized by defects in cobalamin (vitamin B12) metabolism, nervous system development, neurological impairment, intractable epilepsy, and failure to thrive [19]. Functional analysis of *cblX* syndrome has provided a platform whereby the function HCFC1 in discrete organs and tissues can be elucidated. For example, in vitro analysis has demonstrated that HCFC1 regulates metabolism indirectly by regulating the expression of the *MMACHC* gene [12, 14, 15, 19]. These data are further supported by in vivo analysis using transient knockdown in the developing zebrafish [16, 20]. Additional mouse models exist and have demonstrated a function for HCFC1 in diverse cell populations [21, 22], including a subset of NPCs [17]. However, although it is clear that HCFC1 is essential for NPC function [17], previous studies have not yet determined a mechanistic basis for the cellular phenotypes observed. Thus, additional studies examining the function of HCFC1 in NPCs are warranted.

We have created a zebrafish harboring a mutation in the *hcfc1a* gene (*hcfc1a*^co60/+^ allele) using CRISPR/Cas9. Zebrafish have two orthologs of *HCFC1* and in previous studies, we demonstrated that *hcfc1b* is associated with increased NPC production [16]. The two zebrafish paralogs have been shown to have divergent functions, as the knockdown of *hcfc1b* causes facial dysmorphia, but knockdown of *hcfc1a* does not [20]. Therefore, we asked whether germline mutations in the *hcfc1a* gene cause defects in neural development. Our results demonstrate that the *hcfc1a*^co60/+^ allele results in increased numbers of proliferating NPCs (Sox2^+^) and hypomotility. Subsequent RNA sequencing on whole brain homogenates obtained from the *hcfc1a*^co60/+^ allele identified increased expression of *asxl1*, which encodes a transcription factor known to modulate the cell cycle [23, 24]. Furthermore, inhibition of *asxl1* expression in larvae carrying the *hcfc1a*^co60/+^ allele restored the number of NPCs to normal levels. Collectively, our study demonstrates a molecular mechanism by which *hcfc1a* regulates NPC proliferation and brain development.

## Methods

### Experimental model and subject details

The experimental model used in this study is the zebrafish, *Danio rerio*. Zebrafish were obtained from the University of Colorado, School of Medicine or the Zebrafish International Resource Center (ZIRC). The *hcfc1a*^co60/+^ allele was produced using CRISPR/Cas9 methodology as described [25]. The *hcfc1a*^co60/+^ allele was produced at the University of Colorado, School of Medicine by the corresponding author and obtained according to protocols from the University of Texas El Paso. Briefly, a guide RNA (GGTTCATACCAGCCGTTCGT) was designed using publicly available software (ZiFit) [26]. Oligonucleotides from the forward and reverse strand were annealed and ligated into the DR274 vector as described [26]. Guide template DNA was synthesized using PCR amplification with primers (DR274 FWD: TTTGAGACGGGCGACAGAT and DR274 Rev: TTCTGCTATGGAGGTCAGGT) and RNA was synthesized using the MEGAscript T7 in vitro transcription kit. Cas9 was synthesized using the T7 mMessage machine after linearization with PmeI (New England Biolabs) of the Cas9 vector (pMLM3613). A solution (0.2 M KCl with phenol red indicator) containing a final concentration of 500 ng Cas9 and 70 ng of guide RNA was injected at a volume of 2 nl at the single cell stage and embryos were grown to adulthood. The *hcfc1a*^co60/+^ allele was generated from a single founder (F_0_), which was outcrossed with 3 independent wildtype (AB) fish to generate 3 families of F1 carriers. Each family consisted of approximately 20 total fish with equal numbers of males and females. To generate subsequent generations, we outcrossed a minimum of 3 F1 individuals with wildtype (Tupfel Long Fin) to obtain a minimum of 3 families of F2 carriers. We subsequently outcrossed F2 carriers (minimum of 3) with wildtype (AB) fish to produce an F3 generation of approximately 3 total families with equal numbers of males and females. Sanger sequencing confirmed mutation and experiments were initiated in the F3 generation.

The *Tg*(*hsp701*:*HCFC1*) was created using Gateway cloning technology. Briefly, the p5e-*hsp701*, pME-*HCFC1* (created from pcDNA6.1 reported in [20]), p3E-polyA, and the pDestTol2PA were recombined via LR recombination. The resultant vector was co-injected with transposase mRNA synthesized from the pCS2FA vector as previously described [27]. The experiments described herein were performed in the F2 generation, which was produced from a single founder (F_0_). The positive F_0_ carrier was outcrossed with wildtype (AB) to produce 2 families of F1 individuals and a minimum of 3 carriers of the F1 generation were outcrossed to produce 3 families of F2 carriers that were utilized for the experiments described.

For all experiments, embryos (prior to sexual dimorphism) were obtained by crossing AB wildtype, Tupfel Long Fin wildtype, *Tg*(*hsp701*:*HCFC1*), or *hcfc1a*^co60/+^. Experiments were performed at developmental stages only [0–5 days post fertilization (DPF)]. All embryos were maintained in embryo medium at 28 °C. All adults beyond the age of peak breeding age (> 1.5 years) were euthanized using a 10 g/l buffered solution of pharmaceutical grade MS 222 (Tricaine). Fish were emerged in solution for 30 min at room temperature. All euthanized adults underwent secondary euthanasia using a cold ice bath (2–4 °C). Fish were monitored for operculum movement during euthanasia and cessation of movement was indicative of euthanasia. Embryos (< 7 days old) were euthanized using 1–10% sodium hypochlorite solution after being anesthetized in cold ice bath. Prior to fin clipping and before fixation, all fish, adults and larvae, respectively, were anesthetized using MS 222 (150 mg/l for adults and 300 mg/l for embryos). The degree of anesthesia was monitored by operculum movement of adults and cessation of movement for larvae. These techniques are approved and advised by the American Veterinary Medical Association and approved by the University of Texas El Paso Institutional Animal Care and Use Committee.

#### Genotyping

**Fig. 1:**
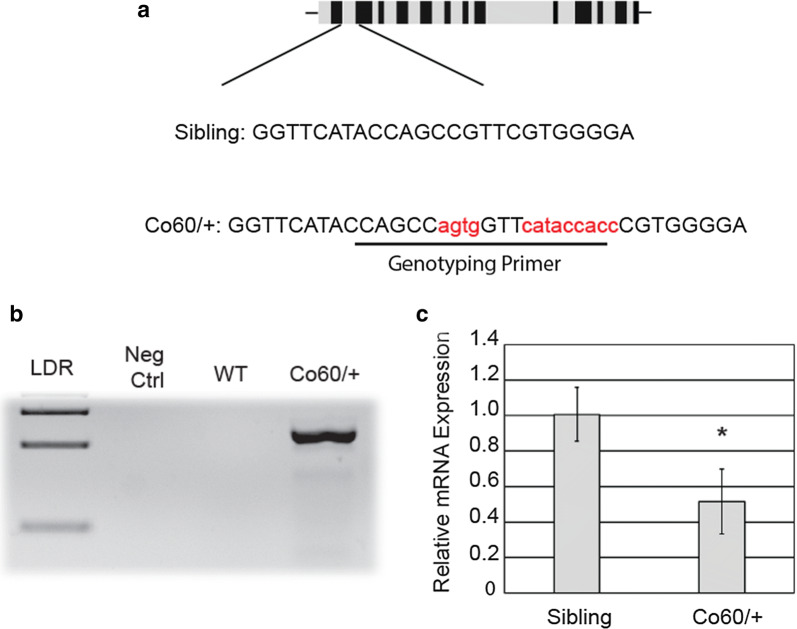
Haploinsufficiency of the *hcfc1a*^*co60/*+^ allele (Co60/+). **a** Schematic of the exon structure of the *hcfc1a* gene (not drawn to scale). The gRNA produced targets the sequence shown within exon 3 (Sibling). The Co60 allele results in an insertion of 13 nucleotides (Red). **b** Primers were designed to specifically amplify the Co60 allele. The specific primer is underlined and bolded in the sequence presented in (**a**). Positive amplification is indicative of heterozygous carriers (Co60/+) with no amplification of the wildtype allele (WT). **c** Quantitative real time PCR analyzing the relative expression of *hcfc1a* in wildtype siblings (Sibling) and the *hcfc1a*^co60/+^ allele (Co60/+) at 2 days post fertilization. N = 16 larvae per group with three biological replicates. *p < 0.05

Genotyping of the *hcfc1a*^co60/+^ allele was performed by lysing excised larval tissue or fin clips (adults) in lysis buffer (10 mM Tris pH 8.2, 10 mM EDTA, 200 mM NaCl, 0.5% SDS, and 200 μg/ml proteinase K) for 3 h at 55 °C. DNA was isolated according to standard phenol chloroform: ethanol precipitation procedures. Primers pairs were developed that specifically bind to and amplify the mutated allele, which did not amplify the wildtype allele. The fragment of interest was amplified by standard PCR at an annealing temperature of 64° (FWD: CCAGTTCGCCTTTTTGTTGT and REV: ACGGGTGGTATGAACCACTGGC). Positive amplification indicates positive carriers of the allele (Fig. 1). Genotyping of the *Tg*(*hsp701*:*HCFC1*) allele was performed with the following primers: forward primer (TGAAACAATTGCACCATAAATTG) present in the *hsp701* promoter and reverse primer in the *HCFC1* open reading frame (CGTCACACACGAAGCCATAG). Amplification indicates the genotype of interest.

#### Immunohistochemistry

Embryos/larvae were fixed in 4% paraformaldehyde (Electron Microscopy Sciences) for minimum of 1 h at room temperature (RT). For each time point, a small piece of caudal tissue was excised for genotyping and the remaining rostral tissue was embedded in 1.5% agarose (Fisher) produced in 5% sucrose (Fisher) and embryo medium. Embedded blocks were incubated overnight in 30% sucrose (Fisher) and then snap frozen with dry ice and cryosectioned (12–20 μM). Sections were washed twice in 1× phosphate buffered saline (PBS) pH 7.4 at RT for 30 min each and blocked for 1 h in blocking buffer (2 mg/ml bovine serum albumin (Fisher), 2% goat serum (Fisher) diluted in 1× PBS). Primary antibody [1:200 anti-Sox2 (Abcam) or 1:500 anti-HuC/D (Fisher)] was incubated overnight at 4 °C and then washed twice in 1× PBS for 30 min each at RT. Alexa fluor antibodies (Fisher) were diluted 1:200 and incubated on each slide for 1 h at RT. All slides were cover slipped using Vectashield (Vector Laboratories) and imaged on a Zeiss LSM 700 at 20×–63× magnification. For cell proliferation, larvae were pulsed in 20 mM 5-ethynyl-2′-deoxyuridine (EdU) (Fisher) diluted in 10% dimethyl sulfoxide (DMSO) (Fisher) for 30 min at RT prior to fixation. EdU was detected using the EdU Click-It technology (Fisher) according to manufacturer protocol.

### Cell quantification

For cell quantification, sections were first divided into forebrain, midbrain, and hindbrain regions using two zebrafish brain atlases; (1) Atlas of Zebrafish Development [28] and (2) Atlas of Early Zebrafish Brain Development [29]. To specify brain regions, major hallmark sub-divisions of each brain section were separated based on age of larvae and the published sections and demarcations present in [29]. For example, sections of the developing brain are organized by letters: A–F indicate forebrain sections, G–L are midbrain, and M-R are hindbrain according to the zebrafish brain atlas. After standardization of the brain region by atlas, cells from each section (12–20 μM) were counted using the ImageJ cell counter. The ImageJ cell counter allows for the manual counting of cells by marking each cell with a colored square and adds the tallied cell to the quantification sheet. The cell counter allows for the tally of 4 independent groups separately with a different color square. Cells were easily visible across all replicates. Only biological replicates with high tissue integrity were quantified. For comparison of representative images, equivalent sections are shown to ensure that minor changes in tissue geometry do not affect the overall conclusion. These sections are standardized from the zebrafish brain atlas. For bar graphs, the average number of cells across each brain region were utilized for quantification using approximately 10–20 equivalent sections/brain region/fish. The number of animals per group is described in each figure legend. To determine the relative increase/decrease in total cell number, the number of total cells/section was divided by the average number of cells present in wildtype siblings for each brain region analyzed and multiplied by 100. All statistical analysis was performed using total numbers of cells/section/brain region. All immunohistochemistry was validated with quantitative real time PCR (QPCR) of each gene analyzed.

#### QPCR and in situ hybridization

Whole mount in situ hybridization (ISH) was performed as described by Thisse and Thisse [30]. Briefly, embryos were harvested and dechorionated at the indicated time point and fixed in 4% paraformaldehyde (PFA) (Electron Microscopy Sciences) for 1 h at RT. Embryos were permeabilized with proteinase K (10 μg/ml) for the time indicated [30]. Permeabilized embryos were prehybridized in hybridization buffer (HB) [50% deionized formamide (Fisher), 5× SSC (Fisher), 0.1% Tween 20 (Fisher), 50 μg/ml heparin (Sigma, St. Louis), 500 μg/ml of RNase-free tRNA (Sigma), 1 M citric acid (Fisher) (460 μl for 50 ml of HB)] for 2–4 h and then incubated overnight in fresh HB with probe (50–100 ng) at 70 °C. Samples were washed, blocked in 2% sheep serum, and incubated with anti-DIG Fab fragments (1:10,000) (Sigma) overnight at 4 °C. Samples were developed with BM purple AP substrate (Sigma) and images were collected with a Zeiss Discovery Stereo Microscope fitted with Zen Software. The *asxl1* cDNA probe sequence was amplified using the following primers: FWD: CATCAACACACGGACCTTTG and REV: CAGTGAGTGGGGTGGAAGTT, purified using a DNA purification kit (Fisher), then ligated into the pGEM-T easy vector using the pGEM T-easy Plasmid Ligation Kit (Promega).

For QPCR, RNA was isolated from embryos at the indicated time point using Trizol (Fisher) according to manufacturer’s protocol. Reverse transcription was performed using Verso cDNA synthesis (Fisher) and total RNA was normalized across all samples. PCR was performed in technical triplicates for each sample using an Applied Biosystem’s StepOne Plus machine with Applied Biosystem’s software. Sybr green (Fisher) based primer pairs for each gene analyzed are as follows: *mmachc* fwd: GCTTCGAGGTTTACCCCTTC, *mmachc* rev: AGGCCAGGGTAGGGTCCTG, *hcfc1a* fwd: ACAGGGCCTAACACAGGTTG, *hcfc1a* rev: TCCTGTGACTGTGCCAAGAG, *asxl1* fwd: CCAGAGCTGGAAAGAACGTC, *asxl1* rev: ACATCTCCAGCTTCGCTCAT, *rpl13a* fwd: TCCCAGCTGCTCTCAAGATT, *rpl13a* rev: TTCTTGGAATAGCGCAGCTT, *sox2* fwd: AACTCCTCGGGAAACAACCA, *sox2* rev: ATCCGGGTGTTCCTTCATGT, *elavl3* fwd: TAACGGCCCTGTCATTAGCA, *elavl3* rev: CGTGTTGATAGCCTTGTCGG, *gfap* fwd: GGCCAACTCTAACATGCAGG, *gfap* rev: ATTCCAGGTCACAGGTCAGG, *olig2* fwd: TTCTGTAGGCCACACACCAG, and *olig2* rev: TTAACTCCGGTGGAGAATCG. Analysis was performed using 2^ΔΔct^.

For RNA sequencing analysis, total RNA was isolated from brain homogenates (N = 12/group from 3 biological replicates), analyzed for RNA integrity, and sequenced at The University of Texas El Paso Border Biomedical Research Center Genomics Core Facility. RNA sequencing was performed in biological triplicate. RNA integrity was assessed with a TapeStation 2200 and the library was prepared with a TruSeq stranded mRNA library preparation kit. Sequencing was performed on a NextSeq 500 (Illumina) using a high output kit V2 (150 cycles). For analysis, the sequences were quality trimmed using Trimmomatic [31] and aligned to the *Danio rerio* genome (build GRCz11) obtained from Ensembl v95 using Tophat2 [32]. Cufflinks [33] was used to determine the differential expression patterns between mutant and wildtype samples.

#### *Tg*(*hsp701*:*HCFC1*) analysis and rescue experiments

F2 carriers of the *Tg*(*hsp701*:*HCFC1*) were incrossed and grown at 28° for 24 h and then split into two groups, non-heat shock and heat shock. Heat shock was performed for 30 min at 38° and then allowed to acclimate at RT for 20 min. Heat shock was initiated at 24 h post fertilization (HPF) and performed every 8 h until 2 or 5 DPF. For LY294002 (Selleck Chemicals) rescue, the drug was dissolved in 100% DMSO (Fisher) and embryos were treated at 24 HPF with a 12uM concentration for a period of 24 h. Media was removed and embryos were dechorionated (if necessary) and fixed for immunohistochemistry.

For morpholino rescue, 2 nl of a 0.1 mM solution of *asxl1* targeting translation inhibiting morpholinos (GTTTGTCCTTCATTTCCTCAGTGTT) or random control morpholinos (Gene-Tools) were injected into offspring of the *hcfc1a*^co60/+^ allele. Injected embryos were fixed at 2 DPF and simultaneously stained for the number of Sox2^+^ and/or EdU+ positive cells. Cells were counted as described above.

### Larval behavioral assay

Embryos were obtained from an outcross of the *hcfc1a*^co60/+^ allele and raised to 5 DPF. Behavioral quantification was performed using the Zebrabox (ViewPoint Behavior Technology) as previously described [34]. Larvae were individually tracked for swim speed and total distance swam in a 96 well plate. The behavioral protocol was a total of 15 min divided into 5-min intervals of dark/light/dark conditions. All larvae were acclimated to the dish and housing conditions for 1 h prior to analysis. Settings for the program include a threshold of 16 and integration period of 300 s. Data was measured as total distance traveled (mm) and total swim speed (mm/s) (Swim Speed = {Total distance traveled in large and small movements) (Smldist + Lardist)}/{Total duration spent by the animal in small and large movements (smldur + lardur)}. Statistical significance was determined according to a T-test. All experiments were performed in biological triplicate.

### Quantification and statistical analysis

For all assays, statistical significance was calculated using a T-test to compare the means of two groups. All assays were performed in biological duplicate and triplicate and all QPCR was performed in technical and biological triplicate or duplicate, respectively. For each assay, the total number of animals (N) is indicated in the figure legend. Number of animals was determined based on power analysis conducted from preliminary studies. For all graphs, statistical significance between groups and the p-value is shown in the figure legend. For cell quantification, the number of Sox2^+^ or EdU+ cells were counted per section and normalized according to the methods section above. All sections were sub-divided based on landmarks in the Atlas of Early Zebrafish Brain Development, 2nd Edition and then separated into specific brain regions (forebrain, midbrain, and hindbrain). All graphs represent error bars as standard error of the mean (SEM).

## Results

### Production of the *hcfc1a*^co60/+^ allele

Previous studies suggest that HCFC1 regulates the number of NPCs in vitro and in mouse models [14–17]. *HCFC1* is highly conserved across species [19] and zebrafish have been used as a model system to understand the mechanisms by which mutations in *HCFC1* cause disease [16, 20]. Therefore, we developed a zebrafish harboring a germline mutation in the *hcfc1a* gene using CRISPR/Cas9 technology. We developed a specific guide RNA (sgRNA) that targets exon 3 of the *hcfc1a* gene (Fig. 1a). The sgRNA was injected at the single cell stage and resulted in the net insertion of 13 nucleotides (Fig. 1a). The introduction of these nucleotides is predicted to introduce a premature stop codon and encode a peptide of 94 amino acids in length (Additional file 1: Figure S1). Full length Hcfc1a is approximately 1778 amino acids in length. Genotyping of the *hcfc1a*^co60/+^ allele was developed according to the materials and methods section, using a reverse primer unique to the mutant allele in the amplification strategy (Fig. 1a). Positive amplification was indicative of positive carriers (Fig. 1b), as the primers did not bind to or amplify the wildtype allele. Initial crosses between heterozygous carriers of the *hcfc1a*^co60/+^ allele failed to generate homozygous progeny and did not obey Mendelian inheritance patterns. These results indicate that Hcfc1a is required for early development, which is consistent with previously published studies [21, 22], however the mechanism for embryonic lethality of the homozygous allele was not explored further here. Importantly, heterozygous carriers survived to adulthood with a lifespan equivalent to wildtype adult zebrafish. Adult heterozygous carriers did not show any gross morphological phenotypes associated with the allele. Larval heterozygous carriers did not show overt morphological phenotypes during early development, which is consistent with previous studies using anti-sense morpholinos targeting the *hcfc1a* gene [20]. However, based on previous studies [14, 15] we surmised that heterozygous carriers of the *hcfc1a*^co60/+^ allele would have defects in overall *hcfc1a* expression and potential defects in brain development. Therefore, we measured the expression of *hcfc1a* in carriers of the *hcfc1a*^co60/+^ allele and their wildtype siblings using QPCR. We designed primers to detect *hcfc1a* expression downstream of Exon 3 that span exons 15 and 17 to ensure that the primers were capable of detecting changes in total mRNA expression and not truncated N-terminal transcripts. As shown in Fig. 1c, at 2 DPF carriers of the *hcfc1a*^co60/+^ allele had a 50% decrease in total *hcfc1a* mRNA (p < 0.05).

### Hcfc1a regulates NPC number in vivo

**Fig. 2:**
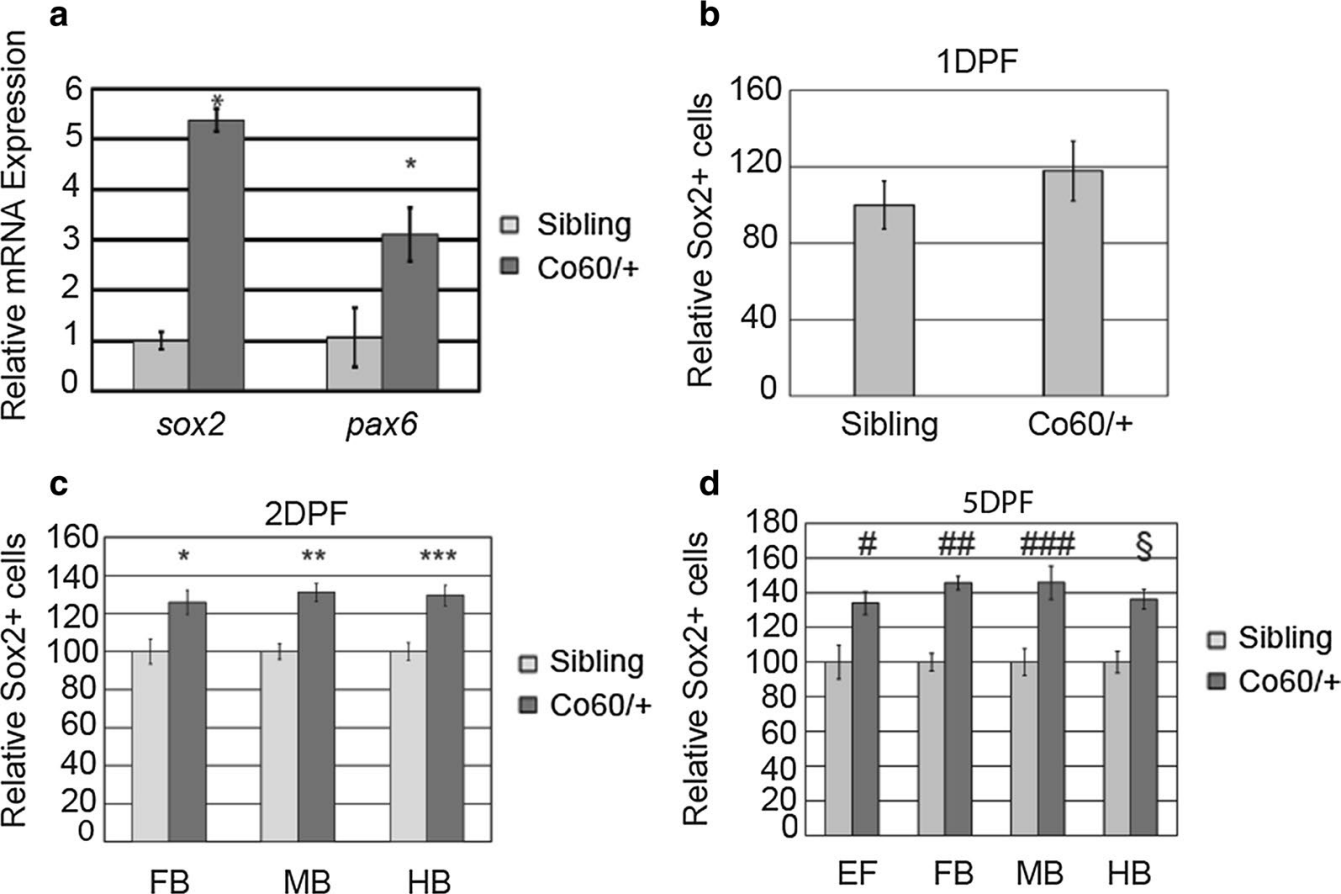
The *hcfc1a*^co60/+^ allele increases the number of Sox2^+^ cells in the developing brain. **a** Quantitative real time PCR analyzing the relative expression of *sox2* and *pax6* in wildtype siblings (Sibling) and the *hcfc1a*^co60/+^ allele (Co60/+). N = 7 larvae per group/biological replicate *p < 0.05. **b**–**d** Quantification of the number of Sox2^+^ cells at 1 day post fertilization (DPF) (**b**), 2 DPF (**c**), and 5 DPF (**d**) in the early forebrain (EF), forebrain (FB), midbrain (MB), and hindbrain (HB). *p = 0.006828, **p = 1.13873E−05, ***p = 0.000107, ^#^p = 0.010168, ^##^p = 4.55E−06, ^###^p = 1.97E−07, and ^§^p = 0.005476. In **b** total number of animals is Sibling (N = 6) and *hcfc1a*^co60/+^ (N = 6), **c** total animals is Sibling (N = 7) and *hcfc1a*^co60/+^ (N = 7), and **d** total animals is Sibling (N = 4) and *hcfc1a*^co60/+^ (N = 4). All error bars represent standard error of the mean

**Fig. 3:**
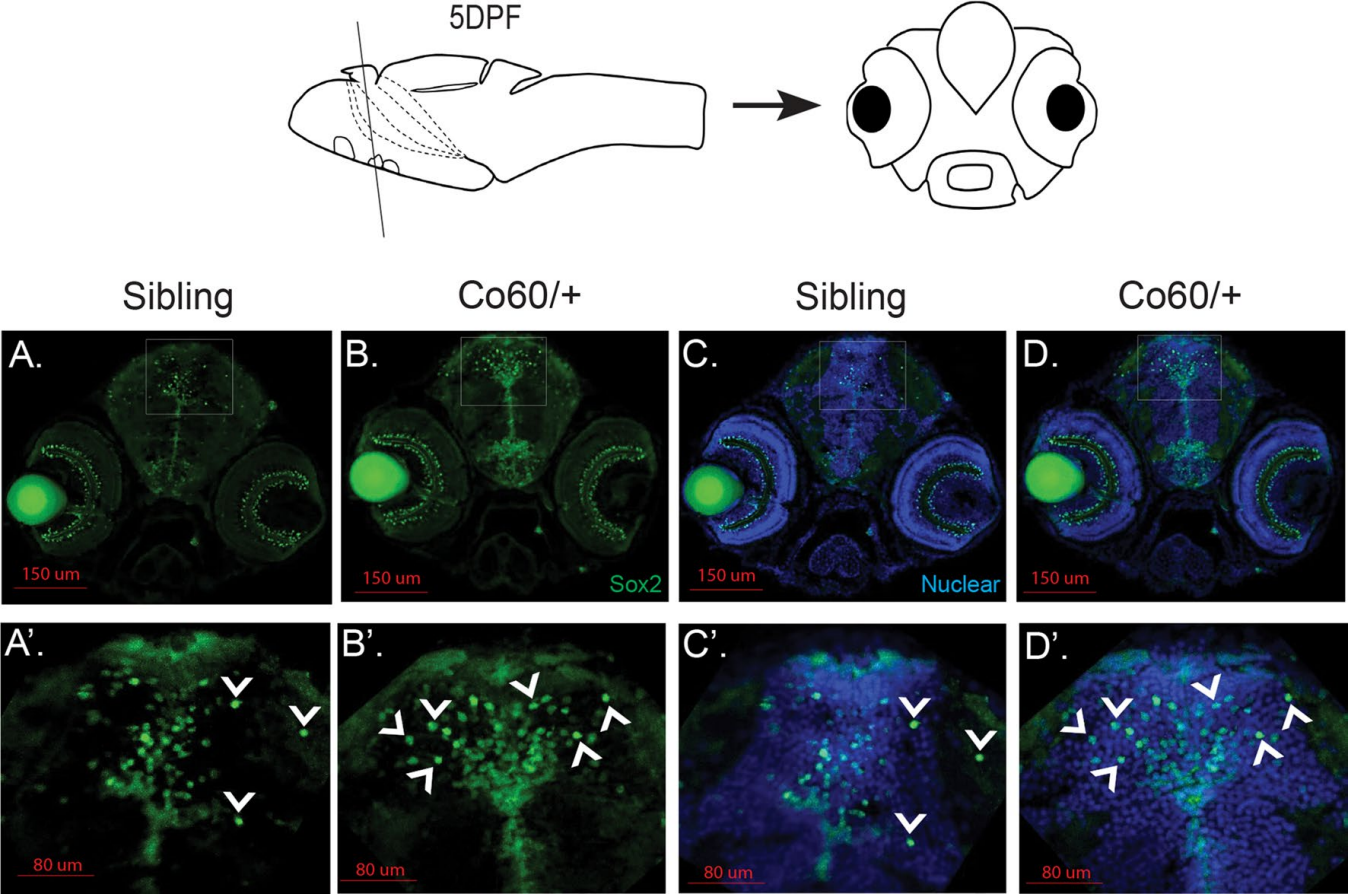
Increased Sox2^+^ cells are present in the *hcfc1a*^co60/+^ allele. **a**–**d**, **a**′–**d**′. Representative images of brain sections from larvae at 5 days post fertilization (DPF) stained with Sox2 antibodies (green) and Hoescht DNA content dye. Whole brain (20×) and the ventricular zone (63×) are shown. Images were captured from sections on a confocal microscope. A schematic of the zebrafish at 5 DPF is shown above representative images with a line to indicate the cross section below

Several studies suggest that mutation or abnormal expression of *Hcfc1* (mouse) mRNA disrupts the number of NPCs, their proliferation, and/or their differentiation. Moreover, we have previously published that *hcfc1a* is expressed in the developing brain across the forebrain, midbrain, and hindbrain [20]. Therefore, we asked whether the decrease in *hcfc1a* mRNA expression in carriers of the *hcfc1a*^co60/+^ allele resulted in abnormal numbers of NPCs in vivo. To test this, we first measured the expression of *sox2* and *pax6* using QPCR at 5 DPF. We used *sox2* and *pax6* as a readout for NPCs because they are co-localized and established markers of NPCs in the field [35–37]. At 5 DPF, the expression of both *sox2* and *pax6* were up-regulated in *hcfc1a*^co60/+^ larvae relative to their wildtype siblings (Fig. 2a). We next compared the total number of Sox2^+^ cells in *hcfc1a*^co60/+^ larvae and their wildtype siblings over the course of development at 1 (Fig. 2b), 2, and 5 DPF. Increased NPCs were not observed until 2 DPF (Fig. 2c), but this increase was sustained until 5 DPF (Fig. 2d). *hcfc1a*^co60/+^ larvae had increased numbers of NPCs in the forebrain, midbrain, and hindbrain regions, with NPCs highly enriched in the ventricular region of the developing brain (Fig. 3a–d and a′–d′, arrowheads indicate cells). There was approximately 25–30% more Sox2^+^ cells per brain region based upon our quantification (Figs. 2, 3).

### The *hcfc1a*^co60/+^ allele disrupts cell proliferation

**Fig. 4:**
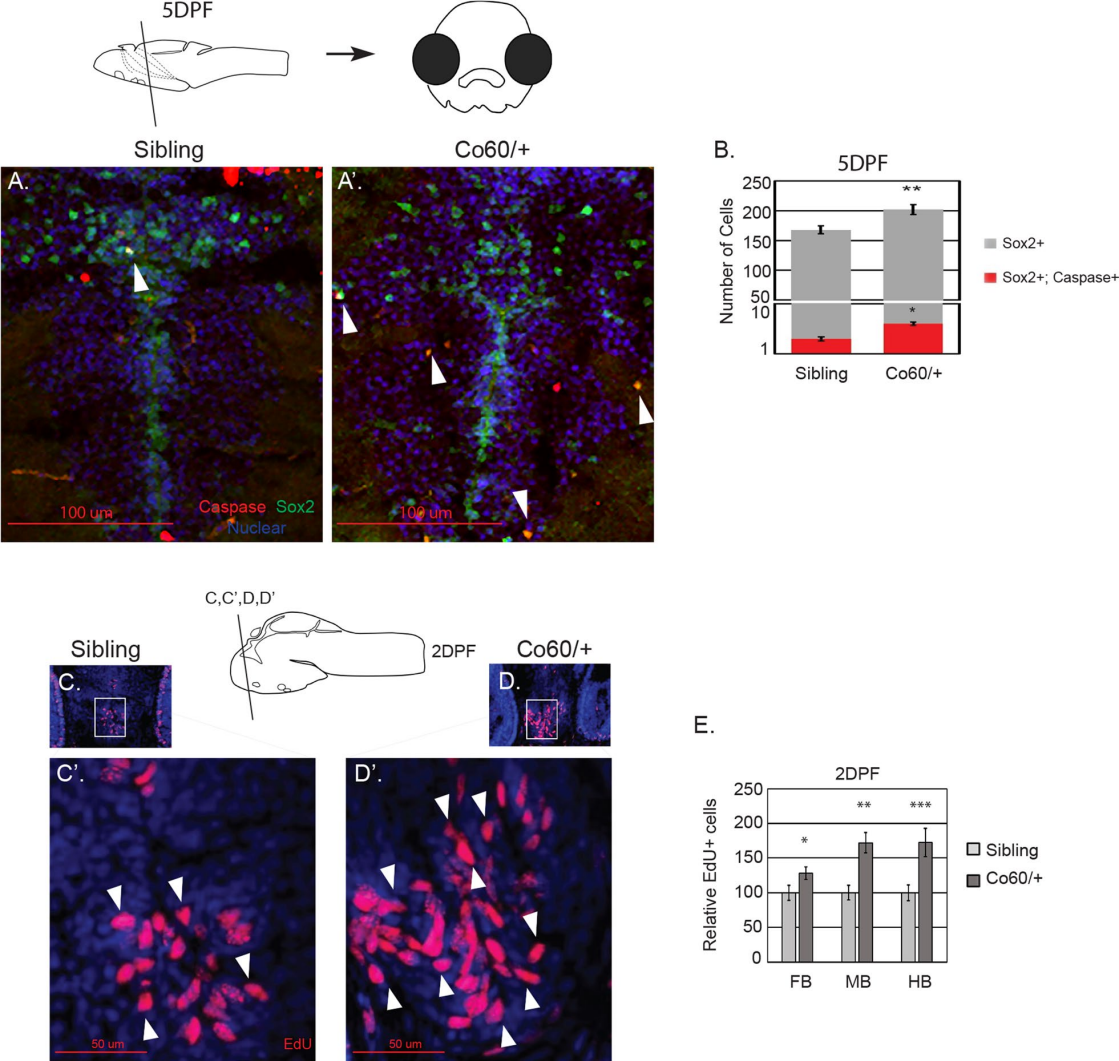
The *hcfc1a*^co60/+^ allele is associated with increased cell proliferation and minimal cell death. **a**–**a**′ Apoptosis of Sox2^+^ neural precursor cells was assessed using anti-caspase 3 and anti-Sox2 antibodies at 5 days post fertilization (DPF). Representative images are depicted from sibling wildtype (Sibling) or the *hcfc1a*^co60/+^ allele (Co60/+). Arrows indicate Sox2^+^ Caspase^+^ neural precursors. **b** The total number of Sox2^+^ Caspase^+^ cells was quantified in wildtype siblings and the Co60/+ allele. Independently, the total number of Sox2^+^ was also quantified and the graph depicts the total number of Caspase positive NPCs (red bars; *p = 1.47822E−16) present within the entire Sox2^+^ population (gray bars; **p < 0.05). Due to the small number of total Sox2^+^ Caspase^+^ cells present, the data is depicted as total numbers of cells in the entire brain. N = 3/group. **c**–**d**, **c**′–**d**′ Representative images of larvae (Sibling or Co60/+) pulsed with ethynyl-deoxyuridine (EdU) to monitor cell proliferation at 2DPF. **c**, **d** Are 20× magnifications and **c**′, **d**′ represent region in the inset at 63× magnification. **e** Quantification of the total number of EdU positive cells in forebrain (FB), midbrain (MB), and hindbrain (HB) of siblings and *hcfc1a*^co60/+^ larvae at 2 DPF. *p = 0.060553, **p = 0.00108, ***p = 0.006642, N = 9 per group. All error bars represent standard error of the mean

Mutation of *Hcfc1* in mice is associated with increased cell death in a subpopulation of NPCs [17], therefore, we hypothesized that the excess NPCs produced undergo cell death. To measure cell death, we performed immunohistochemistry with anti-active caspase 3 antibodies and anti-Sox2 antibodies at 5 DPF. As shown in Fig. 4a, a′, we detected approximately 1–2 Caspase^+^ Sox2^+^ cells in sibling controls (white arrowhead), however larvae harboring the *hcfc1a*^co60/+^ allele had on average approximately 4–5 co-localized cells per/section (white arrowheads in Fig. 4a′). As we detected very few total Caspase^+^ NPCs, we next quantified the total number of NPCs in each group to determine the total number of NPCs surviving. As shown in Fig. 4b, while the *hcfc1a*^co60/+^ allele led to an increase in the number of caspase positive NPCs (red bars), the vast majority of NPCs in both wildtype and *hcfc1a*^co60/+^ larvae were not caspase positive (gray bars), indicating that a significant fraction of NPCs survive. Because of this survival, we next analyzed cell proliferation in *hcfc1a*^co60/+^ larvae and their wildtype siblings using EdU click-it technology. The *hcfc1a*^co60/+^ allele resulted in a statistically significant increase in the number of EdU positive cells in both the midbrain and hindbrain regions (Fig. 4c–d, c′–d′ and quantified in Fig. 4e). We observed an increase in the number of EdU positive cells in the forebrain, although the increase in the forebrain was not significant across multiple biological replicates (p = 0.06). Collectively, these data suggest the *hcfc1a*^co60/+^ allele results in an increase in NPC proliferation, whereby a sub-population of these NPCs undergo cell death, while the majority of those NPCs produced, survive.

### The *hcfc1a*^co60/+^ allele is associated with abnormal expression of pro-neural and pro-glial genes

**Fig. 5:**
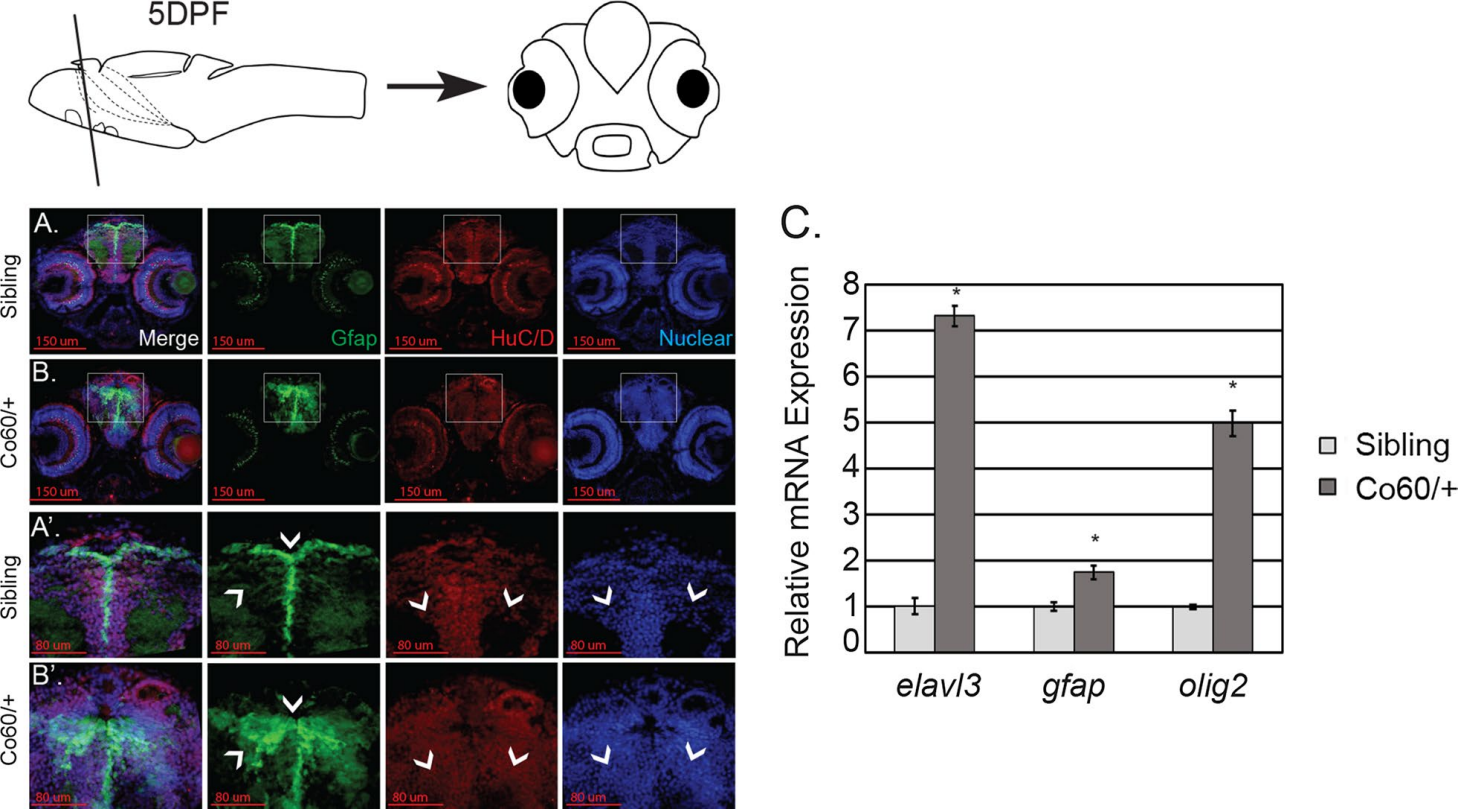
The *hcfc1a*^co60/+^ allele increases the expression of differentiation markers. **a**, **a**′, **b** and **b**′ Representative images of brain sections from *Tg*(*gfap*:EGFP) larvae harboring the *hcfc1a*^co60/+^ allele (Co60/+) at 5 days post fertilization (DPF) stained with Elavl3 (HuC/D) antibodies and Hoescht DNA content dye. 20× representatives of the entire brain (**a**, **b**) and 63× sub-regions (**a**′, **b**′) are shown. Wildtype siblings (N = 6) and *hcfc1a*^co60/+^ larvae (N = 6). Arrowheads indicate regions of increased expression/localization of protein. Arrowheads of Hoescht (Nuclear) images demonstrate regions with cell bodies. Top schematic demonstrates a 5 DPF larval brain with a line to demonstrate the region depicted below. **c** Quantitative real time PCR analysis of the expression of *elavl3*, *gfap*, and *olig2* in siblings and *hcfc1a*^co60/+^ larvae (N = 8/group/biological replicate). Error bars represent standard error of the mean. *p < 0.05

The *hcfc1a*^co60/+^ allele is associated with increased proliferation and an increased number of NPCs. Importantly, the majority of these cells do not undergo cell death and therefore, we asked if the expression of genes associated with either neuronal or glial differentiation was abnormal. We measured the expression of two established markers of neurons and radial glial cells, *elavl3* and *gfap*, by immunohistochemistry. As shown in the Fig. 5a′, b′, the expression of both Gfap and Elavl3 expression was increased in *hcfc1a*^co60/+^ larvae at 5 DPF. Next, we quantified the level of expression of each marker and one additional marker of differentiation (*olig2*) using QPCR at 5DPF. QPCR demonstrated an increase in the level of mRNA expression of each marker (Fig. 5c) in *hcfc1a*^co60/+^ larvae relative to their wildtype siblings (p < 0.05).

### Overexpression of HCFC1 reduces the number of NPCs and decreases neural and glial gene expression

**Fig. 6:**
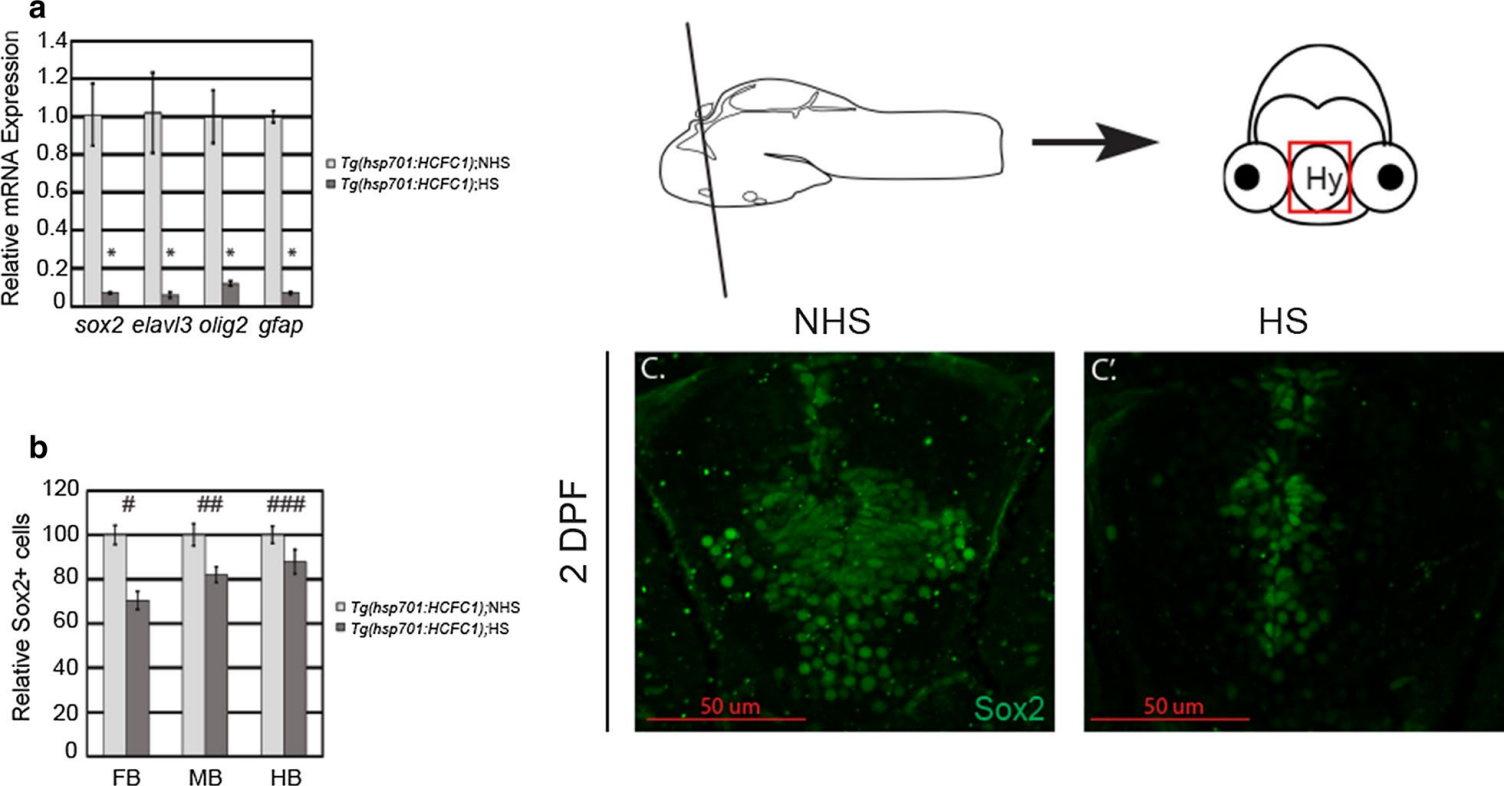
Over expression of HCFC1 decreases NPC number and differentiation. **a***Tg*(*hsp701*: *HCFC1*) were heat shocked according to the methods section and total RNA was isolated from control (No heat shock (NHS)) and heat shock larvae (HS). Quantitative PCR was performed to test the expression of *sox2*, *elavl3*, *olig2*, and *gfap*. Error bars represent standard error of the mean. N = 28/group/biological replicate. *p < 0.05. **b** The total number of Sox2 cells was quantified across forebrain (^#^p = 4.88568E−05), midbrain (^##^p = 0.004359), and hindbrain (^###^p = 0.077). Error bars represent standard error of the mean. N = 5/group. **c**, **c**′ Representative images of 2 days post fertilization (DPF) *Tg*(*hsp701*:*HCFC1*) larvae (NHS or HS) stained with anti-Sox2 antibodies. Schematic demonstrates 2 DPF larval brain section with graphical inset of hypothalamus region (Hy) to specify region shown

Our data demonstrates that haploinsufficiency of *hcfc1a* is associated with increased proliferation and increased numbers of Sox2^+^ cells. Previous studies have shown that over-expression of Hcfc1 (mouse) in vitro is associated with reduced NPC proliferation/growth and reduced NPC proliferation [15]. We tested the effects of HCFC1 over expression in zebrafish by creating a transgenic zebrafish expressing human HCFC1 under the control of the heat shock promoter, *hsp701*. The efficacy of the *hsp701* promoter in zebrafish has been widely established in previous studies [27, 38–42]. We activated expression of HCFC1 by performing a heat shock as described in the methods section for a period of 5 days. We first measured the mRNA expression of *sox2*, *elavl3* (HuC/D), *gfap*, and *olig2* by QPCR. Activation by heat shock of the *Tg*(*hsp701*:*HCFC1*) allele resulted in decreased expression of all markers analyzed (Fig. 6a; p < 0.05). We next analyzed the number of Sox2^+^ cells in the presence and absence of heat shock. Activation of the *Tg*(*hsp701*:*HCFC1*) was associated with a decreased number of NPCs across the forebrain (p = 4.88568E−05), midbrain (p = 0.004359), and hindbrain (p = 0.0776) (Fig. 6b, c–c′).

### Asxl1 is overexpressed in animals with the *hcfc1a*^co60/+^ allele

**Fig. 7:**
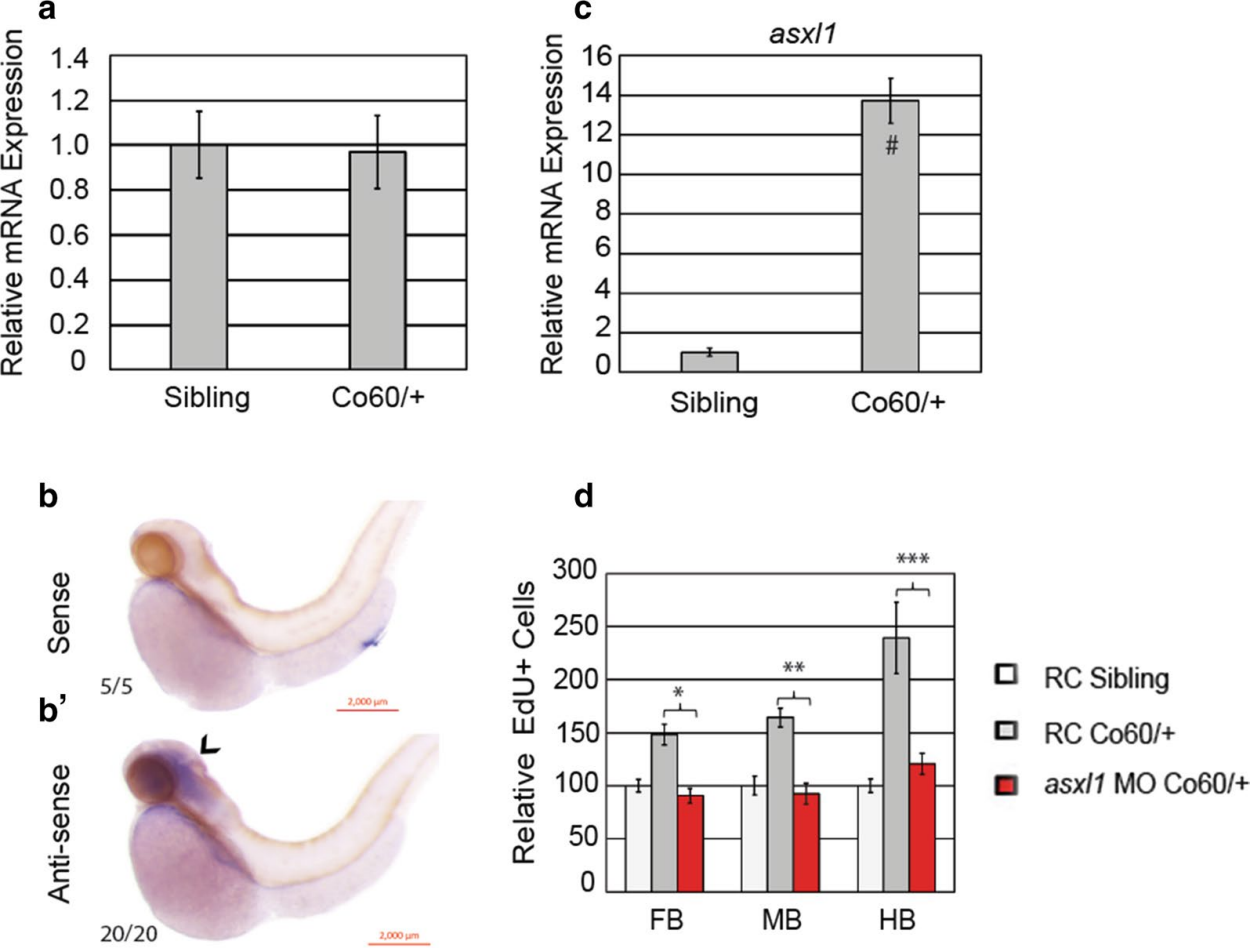
The *hcfc1a*^co60/+^ allele regulates brain development and *asxl1* expression. **a** Quantitative real time PCR (QPCR) analyzing the relative expression of *mmachc* in wildtype siblings (Sibling) and *hcfc1a*^co60/+^ larvae (Co60/+). N = 16 larvae per group with 3 biological replicates. **b**, **b**′ Whole mount in situ hybridization (ISH) was performed at 2 days post fertilization (DPF) using a riboprobe targeting a*sxl1* mRNA. ISH was performed with sense control (**b**) and anti-sense specific probe (**b**′). Purple demonstrates positive staining in the developing brain. N = 20. **c** QPCR analyzing the relative expression of *asxl1* in wildtype siblings (Sibling) and the *hcfc1a*^co60/+^ allele. N = 16 larvae per group with 3 biological replicates. ^#^p < 0.05. **d***hcfc1a*^co60/+^ larvae (Co60/+) and their wildtype siblings (Sibling) were injected with translation inhibiting morpholinos targeting *asxl1* (*asxl1 MO*) or random control (RC) morpholinos. At 2 DPF larvae were pulsed and stained for EdU incorporation and the number of EdU positive cells was counted per brain section. The total number of cells in the forebrain (FB), midbrain (MB), and hindbrain (HB) was calculated. *p = 0.000127, **p = 0.000407, ***p = 0.00712. N = 6/group

HCFC1 is known to regulate metabolism and craniofacial development via the modulation of *MMACHC* expression [14, 19, 20]. Therefore, we hypothesized the neural phenotypes associated with mutations in *hcfc1a* were the direct consequence of defects in *mmachc* expression. We measured the expression of *mmachc* in *hcfc1a* mutants and their wildtype siblings. As shown in Fig. 7a, QPCR analysis demonstrated that *mmachc* expression was unchanged by the *hcfc1a*^co60/+^ allele. Based upon these data, we hypothesized that mutation of *hcfc1a* does not regulate brain development by modulating *mmachc* expression. To better understand the mechanisms downstream of *hcfc1a*, we performed RNA-sequencing at 2 DPF using whole brain homogenates from wildtype and *hcfc1a*^co60/+^ larvae (Additional file 2: Table S1). Using literature analysis, we identified the *asxl1* gene as one possible downstream effector of Hcfc1a in the developing brain. *asxl1* encodes a transcriptional regulator that is essential for proper cell proliferation and whose deletion causes cellular senescence [24, 43]. More importantly, mutations in *ASXL1* have been associated with Boring Opitz Syndrome (605039), which has been characterized by profound intellectual disability [44]. According to in situ hybridization, *asxl1* expression is restricted to the developing zebrafish brain as indicated by the purple stain in panel b′ (Fig. 7b, b′). The sense negative control was absent of this purple stain as shown in Fig. 7b. Additional, QPCR analysis of brain homogenates validated a 14-fold increase of *asxl1* expression at 2 DPF in *hcfc1a*^co60/+^ larvae relative to wildtype siblings (Fig. 7c).

### Inhibition of *asxl1* restores the NPC phenotype in *hcfc1a*^co60/+^ larvae

Deletion of *Asxl1* in mouse embryonic fibroblasts (MEFs) causes growth retardation because Asxl1 regulates the cell cycle via activation of the AKT-E2F axis [24]. Based upon these data, we hypothesized that over-expression of *asxl1* in *hcfc1a*^co60/+^ larvae promotes proliferation of NPCs. To test this hypothesis, we designed a translational blocking morpholino to inhibit *asxl1* expression in *hcfc1a*^co60/+^ larvae. We determined the concentration for injection empirically and selected the highest concentration that promoted > 70% survival of injected embryos. We injected *asxl1* morpholinos or random control morpholinos into *hcfc1a*^co60/+^ larvae and their wildtype siblings at the single cell stage and then analyzed the number of EdU positive cells at 2 DPF. As shown in Fig. 7d, the injection of random control morpholinos into *hcfc1a* mutants and their wildtype siblings had no detrimental effects and recapitulated the NPC phenotype previously observed (i.e. increased NPCs, Figs. 2 and 3). However, the injection of *asxl1* morpholinos completely restored the number of NPCs to wildtype levels in all brain regions (Fig. 7d, red bars).

**Fig. 8:**
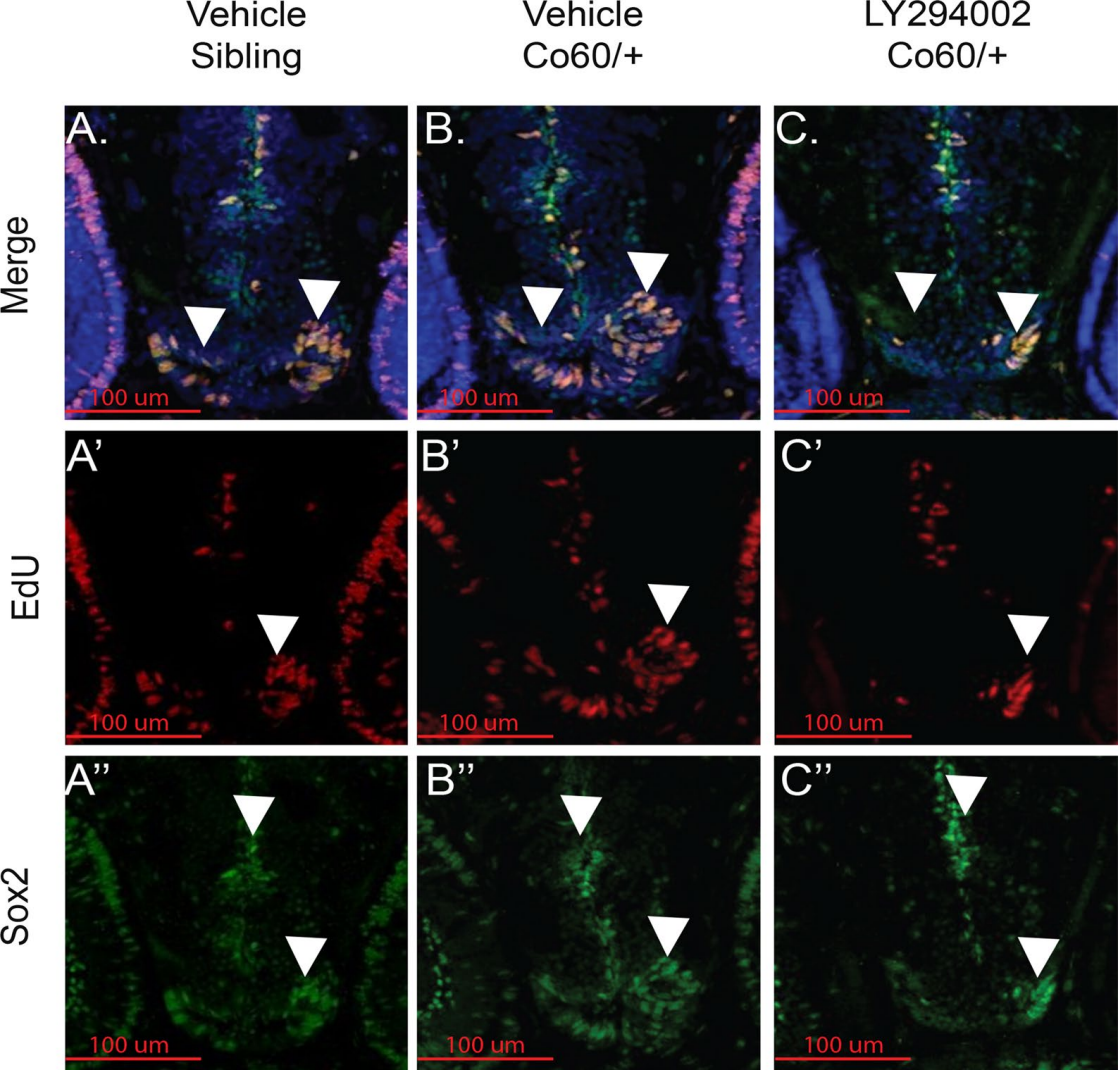
Inhibition of Asxl1 activity restores the NPC deficits in the *hcfc1a*^*co60/*+^ allele. **a**–**a**″, **b**–**b**″ and **c**–**c**″ Representative 20X images of Sibling wildtype (**a**–**a**″), *hcfc1a*^co60/+^ larvae (Co60/+) (**b**–**b**″), or Co60/+ larvae treated with 12 μM LY294002 (**c**–**c**″) at 2 days post fertilization (DPF)

**Fig. 9:**
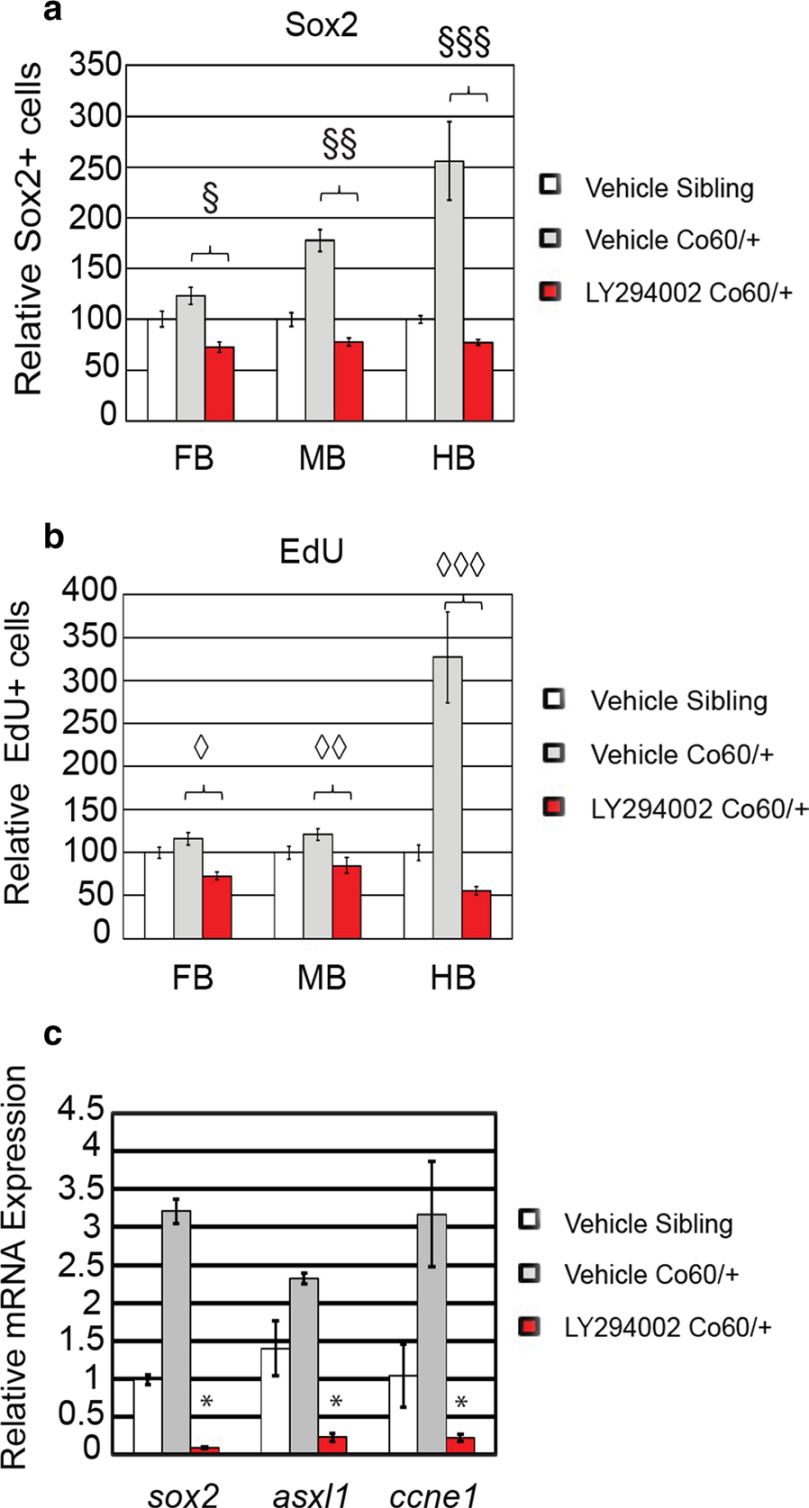
Quantification of the number of NPCs after inhibition of Asxl1 activity. **a**, **b** Quantification of the number of Sox2^+^ (**a**) or EdU (**b**) positive cells in vehicle treated and larvae treated with LY294002 at 2 days post fertilization (DFP). N = 4 Vehicle Sibling, 6 Vehicle *hcfc1a*^co60/+^ larvae, and 4 LY294002 *hcfc1a*^co60/+^ larvae. ^§^p = 0.000168, ^§§^p = 2.08852E−09, ^§§§^p = 0.000153. ^◊^p = 7.22142E−07, ^◊◊^p = 0.000997, ^◊◊◊^p = 0.00014. All error bars represent standard error of the mean. **c***hcfc1a*^co60/+^ larvae and their wildtype siblings (Sibling) were treated at 24 h intervals with 12 μM LY294002 until 5 DPF and then total RNA was isolated from brain homogenates. Quantitative real time PCR was performed to test the expression of *sox2*, *asxl1*, and cyclin E (*ccne1*). N = 10/group/biological replicate. Error bars represent standard error of the mean. *p < 0.05

Morpholinos can display off-target effects [45] and therefore we sought an alternative route of *asxl1* inhibition in *hcfc1a*^co60/+^ larvae. Youn and colleagues have demonstrated that ASXL1 (mouse) expression promotes cell proliferation by binding to AKT kinase and promoting AKT phosphorylation [24]. However, this function can be inhibited with PI3K/AKT inhibitors. Therefore, we treated *hcfc1a*^co60/+^ larvae and their wildtype siblings with LY294002, a PI3K inhibitor as described in [24]. *hcfc1a*^co60/+^ embryos were treated at 24HPF with 12 μM LY294002 or vehicle control (DMSO). Vehicle treatment of wildtype and *hcfc1a*^co60/+^ larvae recapitulated the NPC phenotype (i.e. increased NPCs) present in *hcfc1a*^co60/+^ larvae (Fig. 8a–a″, b–b″ and c–c″, white arrowheads), which was consistent across all brain regions as both the number of Sox2^+^ cells (Fig. 9a) and the number of EdU+ cells (Fig. 9b) were increased in *hcfc1a*^co60/+^ larvae. In contrast, treatment with LY294002 reduced the number of cycling cells (EdU+) and the number of NPCs (Sox2^+^) in *hcfc1a*^co60/+^ larvae, consistent with our hypothesis (Fig. 9a, b, red bars). To complement these data, we next performed mRNA expression analysis of *sox2*, *asxl1*, and cyclin E (*ccne1*) in treated and untreated *hcfc1a*^co60/+^ larvae at 5 DPF. As shown in Fig. 9c, treatment with LY294002 resulted in decreased expression of *sox2* and *asxl1*, which was correlated with decreased *ccne1* expression (p < 0.05).

### Defects in neural development are associated with larval hypomotility

**Fig. 10:**
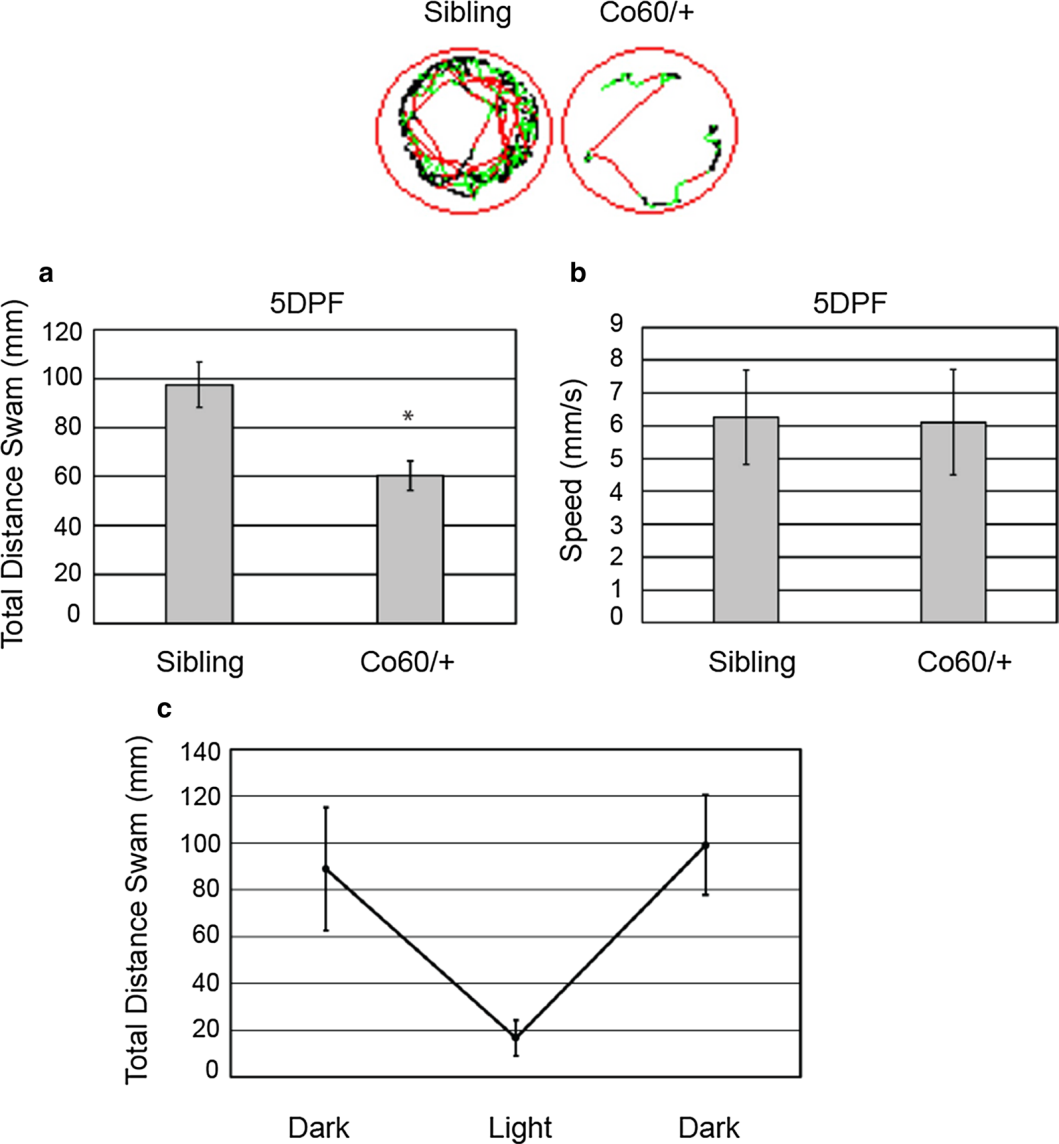
The *hcfc1a*^co60/+^ is associated with hypomotility. Total distance (**a**) and average speed (**b**) of wildtype (Sibling) and heterozygous carriers of the *hcfc1a*^co60/+^ (Co60/+) allele were tracked at 5 days post fertilization (DPF) using ZebraBox technology. Distance and speed were monitored during light stimulus for a 5 min duration. Top panel shows representative tracking patterns from Sibling wildtype and heterozygous carriers of the Co60/+ allele. *p < 0.001. N = 52 Sibling wildtype and 56 Co60/+ individual larvae. **c** At 5 DPF larvae were monitored for total distance swam in alternating dark–light conditions

The functional consequences of the defects in brain development in the *hcfc1a*^co60/+^ allele are not completely understood. However, mutation of *HCFC1* in patients with *cblX* syndrome is associated with movement disorders [19]. Therefore, we performed larval behavioral assays to determine if defects in the number of NPCs were associated with abnormal swim patterns. To test this, we monitored swim behavior of 5 DPF larvae using Zebrabox technology. Carriers of the *hcfc1a*^co60/+^ allele exhibited reduced overall distance swam in response to light stimulus as described in [34], but overall speed was not affected (Fig. 10a, b). These behavioral deficits are consistent with a hypomotility phenotype [46]. Importantly, *hcfc1a*^co60/+^ responded normally to dark–light–dark transitions (Fig. 10c) as has been previously demonstrated [47].

## Discussion

Here we demonstrate that the *hcfc1a*^co60/+^ allele results in an increase in the number of NPCs during early brain development. This increase in NPC number is a direct consequence of over proliferation of NPCs. Mutations in *HCFC1* cause *cblX* syndrome, a multiple congenital anomaly syndrome, associated with cobalamin deficiencies and significant neurological deficits, among other phenotypes [15, 19]. *HCFC1* encodes for a transcriptional co-activator that regulates genes important for metabolism and proliferation [12]. It is suggested that HCFC1 binds to and regulates the expression of > 5000 different genes [18], with various different interacting partners including THAP11 [13] and ZNF143 [48], where mutation of either can cause a *cblX* like disorder [16, 49].

Previous reports suggest that HCFC1 regulates NPC function [14–17]. In vitro, decreased *Hcfc1* (mouse) expression increases the number of NPCs and reduces the expression of markers associated with differentiation [14]. These data are consistent with the known function of *Hcfc1* in cell proliferation [50–52]. Mutation of *Hcfc1* (mouse) is embryonically lethal [21] and consequently, the in vivo function of HCFC1 has been difficult to characterize. Our results are consistent with this observation, as we did not detect viable homozygous larvae or adults. We genotyped larvae from an incross of *hcfc1a*^co60/+^ adults at 2, 4, and 8 HPF and even at the earliest time points, we detected few to zero homozygous larvae. We did not explore this mechanism further, however in previous studies the murine Hcfc1 mutant allele was subject to compensatory mechanisms and only the paternally inherited allele was viable [21]. Zebrafish do not have sex chromosomes, but future studies are warranted that characterize the inheritance of the *hcfc1a*^co60/+^ allele as well as the embryonic lethality.

Recently a cell-type specific mutant allele was created in which *Hcfc1* (mouse) was deleted from a subpopulation of neural precursors (NKX2.1+). This cell type specific deletion of *Hcfc1* (mouse) induces cell death and defects in differentiation without affecting proliferation. These results differ from in vitro assays, whereby decreased *Hcfc1* (mouse) results in an increase in the number of precursors. The discrepancy between these results might be explained by many factors, including the propagation of neurospheres in vitro and the inability to decipher how the developing microenvironment affects normal physiology. However, some studies have helped to shed light on the latter explanation because the in vivo knockdown of *hcfc1b*, one of the zebrafish orthologs of *HCFC1*, resulted in increased NPC proliferation [16]. Thus, the function of HCFC1 is complex, with several unknown cell-type specific functions that can be affected by the surrounding microenvironment.

Here we developed a zebrafish harboring a germline mutation in the *hcfc1a* gene. We analyzed the effects of this mutation on NPC number and proliferation. Consistent with the literature [14–16], our allele resulted in an increase in the number of Sox2^+^ cells and increased cellular proliferation without significant deficits in cell survival. Increased cell proliferation was observed across all brain regions. However, our studies were limited to detection of NPCs using the Sox2 marker. A significant fraction of these cells co-localize with EdU positive cells in the Co60/+ allele, but not all of them. These data strongly suggest that *hcfc1a* is important for the proliferation of Sox2 positive NPCs. However, the NPC population is heterogeneous in nature and can include Sox2^+^ and Sox2^−^ cells. We do not yet understand the effects of *hcfc1a* mutations on the Sox2^−^ population or other NPC sub-populations (actively proliferating versus not proliferating). However, we did observe increased *pax6* expression. *Sox2* and *Pax6* are known to be co-expressed in a subpopulation of NPCs so it is possible these two markers label similar populations of NPCs [37].

The increased numbers of NPCs in the Co60 allele was not associated with increased apoptosis of NPCs, but instead was associated with increased expression of markers associated with neurons and glia. The effects of this increased expression are yet to be elucidated, however studies that provide information on brain volume and the ratio of gray and white matter in the central nervous system are warranted; particularly given that the increases in neurons and glia we observed are unique from the differentiation defects observed in previous studies [14, 17]. However, we suspect the difference in phenotype between previous studies and our own is likely associated with the type of mutation introduced, the brain microenvironment, the cell population analyzed, and the region of the brain of interest. For example, Minocha and Herr [17] deleted exons 2 and 3 of *Hcfc1* using a Cre-Lox system with a cell type specific promoter. The resulting approach introduces the formation of a truncated protein, whereas, our system results in decreased overall expression (Fig. 1), which may be more consistent with previous in vitro assays and haploinsufficiency. However our haploinsufficient allele advances the field because unlike the previous in vitro assays [14, 15], our allele accounts for the broad expression of Hcfc1 which was recently documented by Minocha and colleagues [17].

HCFC1 regulates a myriad of downstream target genes [12, 18] and therefore, the mechanisms by which HCFC1 regulates NPC function are not clear. The majority of the literature focuses primarily on the function of HCFC1 at the *MMACHC* promoter in the human syndrome, *cblX*. *cblX* disorder is the result of mutations in the *HCFC1* gene and these mutations disrupt protein function causing a decrease the expression of *MMACHC* and a metabolic disorder [14, 19]. Interestingly, mutations in *MMACHC* cause *cblC* disorder, which has many overlapping phenotypes with *cblX* including neurodevelopmental defects [53]. These data led us to hypothesize that HCFC1 regulates NPC function by modulating *MMACHC* expression. However, the *hcfc1a*^co60/+^ allele did not disrupt *mmachc* expression. However, whether our allele causes other metabolic deficits is not known and was not explored further here. For example, mutation of the human *HCFC1* gene has been associated with non-ketotic hyperglycinemia [54]. Collectively the data presented here suggests that the *hcfc1a*^co60/+^ allele disrupts NPC proliferation by a novel molecular mechanism. In addition, these data suggest some divergent function between *hcfc1a* and *hcfc1b*, as the latter has been shown to regulate *mmachc* expression and craniofacial development [20].

Our results strongly suggest an *mmachc* independent mechanism underlying the neural developmental phenotypes associated with the *hcfc1a*^co60/+^ allele, but we cannot completely rule out that *hcfc1b* regulates brain development via *mmachc* expression or that other factors including cobalamin, homocysteine, or methylmalonic acid accumulation. However, RNA-sequencing of brain homogenates provided us a list of potential downstream effectors of *hcfc1a*. Of those candidates, the *asxl1* gene was afforded high priority because of its known role regulating cellular proliferation [24, 43, 55] and for its documented function in mouse embryonic stem cells and neural differentiation [23]. Interestingly, the *hcfc1a*^co60/+^ allele causes a 14-fold induction of *asxl1* expression. In mouse embryonic fibroblasts, the deletion of *Asxl1* causes cellular senescence. We observed increased cellular proliferation and therefore, postulated that an increased level of Asxl1 protein was promoting NPC proliferation in *hcfc1a* mutant larvae. Consistent with the known function of *asxl1* and our hypothesis, knockdown of *asxl1* in *hcfc1a* mutant larvae restored the defects in cellular proliferation, resulting in normal numbers of Sox2^+^ cells.

Interestingly, *ASXL1* has been shown to regulate cell proliferation in other cell types [55, 56] and this activity has been associated with activation of AKT. Based upon these data, we attempted to restore the phenotypes present in the *hcfc1a*^co60/+^ allele by inhibiting ASXL1 activity downstream of PI3K [24]. Consistent with this role, the inhibition of ASXL1 activity using pharmacological inhibition completely restored the NPC deficits present in *hcfc1a* mutants. Thus, our data suggest a mechanism whereby *hcfc1a* regulates the expression of Asxl1 and the cell cycle during early brain development. However, whether *hcfc1a* regulates *asxl1* by directly binding to the *asxl1* promoter is still not known, but interestingly ASXL1 and HCFC1 interact with one another in myeloid cells to regulate proliferation and differentiation [57]. Thus, these two proteins may regulate the activity of one another at multiple levels.

We observed changes in the expression of various markers of neurons and glia. However, the physiological consequences of these changes are not currently known. Zebrafish have emerged as a model for neurodevelopmental disorders [58] and behavioral assays for seizure [59] and motor deficits [60] have been described. Therefore, we characterized the locomotion of *hcfc1a* mutants in response to light stimulus [61]. Our results, using Zebrabox technology, demonstrated reduced motility as indicated by decreased distance travelled. Importantly, the decreased motility during a light stimulus was not due to an overall defective response to light, as our analysis demonstrated that carriers of the *hcfc1a*^co60/+^ allele responded to normally to light stimulus, as indicated by the “V” like pattern in dark–light–dark conditions [62]. Decreased distance travelled has been previously defined as hypolocomotion and is associated with motor incoordination [63]. Hypolocomotion has been demonstrated in zebrafish models of ALS [60, 64] and fetal akinesia [65], two disorders characterized by motor deficits. Interestingly, the hypolocomotion we observed was correlated with increased NPCs and defects in the expression of various markers associated with differentiation. We did not observe short convulsions or whirlpool like behaviors in mutant larvae, which would have been indicative of a seizure phenotype and it is likely that the protocol we used to detect behavioral deficits does not stimulate a seizure like phenotype. Future studies that use multiple stimuli, including low dose convulsants will likely shed light on the epileptic phenotypes associated with mutations in *hcfc1a*.

## Conclusions

Our study focuses on the function of *hcfc1a*, one ortholog of *HCFC1*, during brain development. Specifically, we focus on the function of *hcfc1a* in modulating NPC number and proliferation. We demonstrate that HCFC1 is essential for the proliferation of NPCs. Importantly we connect these cellular deficits to a molecular mechanism whereby *hcfc1a* indirectly or directly regulates *asxl1* expression to control cellular proliferation. Thus, we propose that our system has the potential to inform about the transcriptional program regulating NPC function.

## Supplementary information

**Additional file 1: Figure S1.** The Co60 allele is predicted to produce a premature stop codon. The predicted protein would cause an N-terminal truncation. ClustalOmega alignment of the allele encoded protein and the full length Hcfc1a is depicted.

**Additional file 2: Table S1.** RNA-sequencing reveals 36 upregulated and downregulated genes.

## Data Availability

The RNA-sequencing data sets generated during this study have been deposited into the GEO database with Accession number GSE132864 and a summary is included within the article. All files are also available from the corresponding author upon request. All files are accessible at the following link: https://www.ncbi.nlm.nih.gov/geo/query/acc.cgi?acc=GSE132864 and available as of May 6, 2020.

## Abbreviations

NPC: Neural precursor
cblX: Methylmalonic acidemia and homocysteinemia, cblX type
F_0_: Founder
DPF: Days post fertilization
HPF: Hours post fertilization
RT: Room temperature
DMSO: Dimethylsulfoxide
QPCR: Quantitative real time PCR
ISH: Whole mount in situ hybridization
SEM: Standard error of the mean
EdU: 5-ethynyl-2′-deoxyuridine
mM: Millimolar
μM: Micromolar
PFA: Paraformaldehyde
SSC: Saline sodium citrate
DIG: Digoxigenin
AP: Alkaline phosphate
CRISPR/Cas9: Clustered Regularly Interspaced short palindromic repeats/Cas9 nuclease
ng: Nanograms
nl: Nanoliters
EDTA: Ethylenediaminetetraacetic acid
NaCl: Sodium chloride
SDS: Sodium dodecyl sulfate
μg: Microgram
PCR: Polymerase chain reaction
HB: Hybridization buffer
MEF: Mouse embryonic fibroblasts
