# Supplementary material for: Hcfc1a regulates neural precursor proliferation and asxl1 expression in the developing brain
Source: BMC Neurosci. 2020 Jun 10;21:27. doi: 10.1186/s12868-020-00577-1 (PMC7288482; doi:10.1186/s12868-020-00577-1)
Supplement: Supplementary file 1 — Additional file 1: Figure S1. The Co60 allele is predicted to produce a premature stop codon. The predicted protein would cause an N-terminal truncation. ClustalOmega alignment of the allele encoded protein and the full length Hcfc1a is depicted. [file 12868_2020_577_MOESM1_ESM.docx]

Additional Figure S1

CLUSTAL O(1.2.4) multiple sequence alignment

hcfc1a MTSPGVPGTTGAPLQPRWKRVLGWSGPVPRPRHGHRAVAIKELMVVFGGGNEGIVDELHV 60

Co60 MTSPGVPGTTGAPLQPRWKRVLGWSGPVPRPRHGHRAVAIKELMVVFGGGNEGIVDELHV 60

************************************************************

hcfc1a YNTATNQWFIPAVRGDIPPGCAAY---GFVCDGTRLLVFGGMVEYGKYSNDLYELQASRW 117

Co60 YNTATNQWFIPASGSYHPWGHPSRLCGLWFCV---------------------------- 92

************ . * * : :.*

hcfc1a EWKKLKPKAPKNGVPPCPRLGHSFSLVGNKCYLFGGLANDSEDPKNNIPRYLNDLYTLEL 177

Co60 ------------------------------------------------------------ 92

hcfc1a RPGSSVAGWDVPVTYGVLPPPRESHTAVIYTEKVTKKSRLVIYGGMSGCRLGDLWTLDID 237

Co60 ------------------------------------------------------------ 92

hcfc1a TLTWNKPAISGAAPLPRSLHSATTITNKMYVFGGWVPLVMDDVKVATHEKEWKCTNTLAC 297

Co60 ------------------------------------------------------------ 92

hcfc1a LNLDTLAWETVLMDTLEDNIPRARAGHCAVAINNRLYIWSGRDGYRKAWNNQVCCKDLWY 357

Co60 ------------------------------------------------------------ 92

hcfc1a LETERPNPPSRVQLVRANTNSLEVSWGAVSTADTYLLQLQKYDIPAATAATSPALNAAPS 417

Co60 ------------------------------------------------------------ 92

hcfc1a LPGNSPKSPAPAAAAPSAQSLPHSGITSVPQAASPTASVLPGTPASPLAASMARGPAILK 477

Co60 ------------------------------------------------------------ 92

hcfc1a VAAPQSGTGASIVTVRQASQVGKSPVTVASLPAGVRMVVPSQTAQGTIGSSPQMSGMAAL 537

Co60 ------------------------------------------------------------ 92

hcfc1a AAAAAATQKIPPSPGATVLNMPAGATIVKTVAVTPGASTLPTTVKVASPLMVSNPATRML 597

Co60 ------------------------------------------------------------ 92

hcfc1a KTAAAQVATPTVSSPTTGARPIITVHKSGTVTVAQQAQVVTTVVGGVTKTITLVKSPLSM 657

Co60 ------------------------------------------------------------ 92

hcfc1a GGNLISNLGKVVSVVQNKPVQSATVTGQASNPLTQIIQTKGPLPPGTILKLVTSADGKPT 717

Co60 ------------------------------------------------------------ 92

hcfc1a TIITTSQAGGTGNKPTILGISGMSPTSTSKPGTTTIIKTIPMSAVQQGAAGVTSTTGVKS 777

Co60 ------------------------------------------------------------ 92

hcfc1a PITIITTKVMTSGTPGKIITAMPKIGTAAGQQGLTQVVLKGAPGQPGTILRTVPMGGVRL 837

Co60 ------------------------------------------------------------ 92

hcfc1a VSPVSGVKPTVTTLVVKGTTGVTTLGTVTGTVSSSLAGGSLASANATLATPITTLGTIAT 897

Co60 ------------------------------------------------------------ 92

hcfc1a LASQVINATNVTAAQSNLTTVTSTMQPTQVTLITTPSGVEAQPGQDLPVSFLASPTSEQP 957

Co60 ------------------------------------------------------------ 92

hcfc1a TSTESGTGEASGSVTLVCSNPPCETHETGTTNTATTATSNMGAGPAGTVQRVCSNPPCET 1017

Co60 ------------------------------------------------------------ 92

hcfc1a HETGTTNTATTASANMGAVQRVCSNPPCETHETGTTSTTTTASSNMGTAPAGTVQRVCSN 1077

Co60 ------------------------------------------------------------ 92

hcfc1a PPCETHETGTTNTATTASSNMGGNQPGAVQRVCSNPPCETHETGTTNTATTASSSMGGGQ 1137

Co60 ------------------------------------------------------------ 92

hcfc1a EGTVQRVCSNPPCETHETGTTNTATTSSSNMGGDQAGAVQRVCSNPPCETHETGTTNTAT 1197

Co60 ------------------------------------------------------------ 92

hcfc1a TATCNMETDEGTAAQRGAITTVTQATPLPGPAVPSISSITESSSEAGAEPVVMESSEAES 1257

Co60 ------------------------------------------------------------ 92

hcfc1a LQTEGQAEAEAVAMQAEFQTEAGAVALPTDFQEDAAALPADFQGEAVAVAMQAESQADAV 1317

Co60 ------------------------------------------------------------ 92

hcfc1a PMEQDVSGMAEGEAAQEQLPTAEMEDVDAASASQAAVLALPPELMADGQSTTLMVTGLTP 1377

Co60 ------------------------------------------------------------ 92

hcfc1a EELAVTAAAEAAAQAAATEEAQALAIQAVLQAAQQAVMSDAGGDDQQTHTIPIVLTQQEL 1437

Co60 ------------------------------------------------------------ 92

hcfc1a AALVQQQQQLQAAQQQAAAQAALPTEGLAPADSLNDPLSESNGHNEMAAAATSAVVTLLP 1497

Co60 ------------------------------------------------------------ 92

hcfc1a RTAAETLAPSSTLAPVVVASPAKMQAAAALTEVANGIEAGKQNPPTVTVKPQVKKENQWF 1557

Co60 ------------------------------------------------------------ 92

hcfc1a DVGIVKVTNTVVTHFYMPADDSAYVENDSGTAPDYSQMKRVELQPGTAYKFRVAGINTCG 1617

Co60 ------------------------------------------------------------ 92

hcfc1a RGTFSEISAFKTCLPGFPGAPCAIKISKSPDGAHLTWEPPSVTSGKITEYSVYLAIQSSQ 1677

Co60 ------------------------------------------------------------ 92

hcfc1a TTEAKPSAPAQLAFMRVYCGPNPSCLVQSSSLSNAHIDYTTKPAIIFRIAARNEKGYGPA 1737

Co60 ------------------------------------------------------------ 92

hcfc1a TQVRWLQESSKDGLSAKPAPKRAVSSPDTKGIGQKKARMDQ 1778

Co60 ----------------------------------------- 92

Figure Legend: The Co60 allele is predicted to produce a premature stop codon. The predicted protein would cause an N-terminal truncation. ClustalOmega alignment of the allele encoded protein and the full length Hcfc1a is depicted.
