# Supplementary material for: Hcfc1a regulates neural precursor proliferation and asxl1 expression in the developing brain
Source: BMC Neurosci. 2020 Jun 10;21:27. doi: 10.1186/s12868-020-00577-1 (PMC7288482; doi:10.1186/s12868-020-00577-1)
Supplement: Supplementary file 2 — Additional file 2: Table S1. RNA-sequencing reveals 36 upregulated and downregulated genes. [file 12868_2020_577_MOESM2_ESM.docx]

| **Gene Name** | **Gene ID** | **Fold Change** |
| --- | --- | --- |
| si:dkey-222f8.6 | XLOC_021011 | 14.4930988 |
| asxl1 | XLOC_016905 | 3.85582801 |
| ccl44 | XLOC_003025 | 3.76589623 |
| zgc:112970 | XLOC_030718 | 3.45845584 |
| si:dkey-15j16.6 | XLOC_014036 | 2.66543586 |
| mucms1 | XLOC_001109 | 2.59007221 |
| CR388052.1 | XLOC_023401 | 2.44522307 |
| si:dkey-15j16.3 | XLOC_014035 | 2.39033346 |
| CABZ01017723.1 | XLOC_031170 | 2.23752184 |
| cart3 | XLOC_028234 | 2.10261498 |
| SLC22A3 | XLOC_009181 | 1.97809228 |
| camk1gb | XLOC_017012 | 1.91222151 |
| vtg1,vtg4,vtg5,vtg6,vtg7 | XLOC_016435 | 1.91072755 |
| si:dkey-208m12.2 | XLOC_016485 | 1.88711718 |
| usp48 | XLOC_003342 | 1.88552791 |
| si:dkey-85k7.7 | XLOC_027050 | 1.86192429 |
| znf319 | XLOC_018986 | 1.75290426 |
| si:dkey-14o1.18 | XLOC_029699 | 1.73919114 |
| cry-dash | XLOC_018109 | 1.68379868 |
| si:ch73-60p2.1 | XLOC_016151 | 1.67438066 |
| hbbe3 | XLOC_004139 | 1.64033724 |
| c3b.1,c3b.2 | XLOC_016458 | 1.53598227 |
| hcfc1a | XLOC_003246 | 0.68365388 |
| fut9d | XLOC_028523 | 0.6648173 |
| si:ch211-183d21.1 | XLOC_017905 | 0.58211004 |
| exosc3 | XLOC_031084 | 0.55764572 |
| si:dkey-147f3.4 | XLOC_016723 | 0.55397064 |
| cox6b1 | XLOC_018338 | 0.54151104 |
| BX530067.1 | XLOC_011301 | 0.53422498 |
| si:dkey-57c15.9 | XLOC_016192 | 0.50905352 |
| si:dkey-9l20.3 | XLOC_016618 | 0.47144985 |
| slc23a3 | XLOC_030187 | 0.34691461 |
| wu:fi09b08 | XLOC_031057 | 0.34631257 |
| hsp70l | XLOC_028453 | 0.27760694 |
| si:dkey-51a16.10 | XLOC_004659 | 0 |

**Table S1: RNA-Sequencing reveals 36 upregulated and downregulated genes.**

Illumina Sequencing was performed on triplicates of whole brain homogenates from 2dpf *hcfc1a*+/^co60^ allele zebrafish larvae (N=12). Analysis revealed 23 upregulated (Green cells) and 14 downregulated (Red Cells) genes with statistical significance of 2.5 x 10 ^-5^ or greater.
